# *De Novo* Human Cardiac Myocytes for Medical Research: Promises and Challenges

**DOI:** 10.1155/2017/4528941

**Published:** 2017-02-20

**Authors:** Veronique Hamel, Kang Cheng, Shudan Liao, Aizhu Lu, Yong Zheng, Yawen Chen, Yucai Xie, Wenbin Liang

**Affiliations:** ^1^University of Ottawa Heart Institute & Department of Cellular and Molecular Medicine, University of Ottawa, Ottawa, ON, Canada; ^2^Department of Cardiology, Xi'an No. 3 Hospital, Xi'an, China; ^3^Department of Cardiology, Xi'an Central Hospital, Xi'an, China; ^4^Department of Anesthesiology, Zhujiang Hospital, Southern Medical University, Guangzhou, China; ^5^Department of Cardiology, The Second Affiliated Hospital, Zhejiang University School of Medicine, Hangzhou, China; ^6^Department of Cardiology, Shanghai Ruijin Hospital, Shanghai Jiaotong University School of Medicine, Shanghai, China

## Abstract

The advent of cellular reprogramming technology has revolutionized biomedical research.* De novo* human cardiac myocytes can now be obtained from direct reprogramming of somatic cells (such as fibroblasts), from induced pluripotent stem cells (iPSCs, which are reprogrammed from somatic cells), and from human embryonic stem cells (hESCs). Such* de novo* human cardiac myocytes hold great promise for* in vitro* disease modeling and drug screening and* in vivo* cell therapy of heart disease. Here, we review the technique advancements for generating* de novo* human cardiac myocytes. We also discuss several challenges for the use of such cells in research and regenerative medicine, such as the immature phenotype and heterogeneity of* de novo* cardiac myocytes obtained with existing protocols. We focus on the recent advancements in addressing such challenges.

## 1. The Need for Human Cardiac Myocytes in Biomedical Research

Despite extensive research, heart disease remains the number 1 killer worldwide leading to >17.3 million deaths in 2013, accounting for 31% of all cause deaths [1]. The economic burden for heart disease is estimated to be US$863 billion in 2010 and expected to increase to US$1,044 billion in 2030 [2]. On the other hand, market withdrawal of new drugs due to unexpected cardiotoxicity significantly contributes to the rising costs for drug developments [3]. One of the major reasons is that direct studies in human patients or healthy volunteers are limited by ethical concerns, and it is difficult to obtain and maintain primary human cardiac myocytes from patients for* in vitro* experiments. Accordingly, most heart research has been performed with experimental animal models and cultured animal cells. These animal studies are useful, but their values to understand human heart disease are compromised by profound species difference, such as in the composition of ion channels within the myocardial tissue [4], as well as differences in the size and beating rate of the hearts (500 beats/min in mouse versus 70 beats/min in human). Therefore, human cardiac myocytes that can be readily obtained without ethical limitations are needed to boost the fidelity of biomedical research.

## 2. Methods for Generating* De Novo* Human Cardiac Myocytes

Last decades have seen exciting advancements in biotechnology that make it possible to generate* de novo* human cardiac myocytes from embryonic stem cells (ESCs), induced pluripotent stem cells (iPSCs), or somatic cells via direct cellular reprogramming. Here, we provide a brief review on this topic. For more details, the readers are referred to recent excellent reviews [5, 6].

### 2.1. Cardiac Differentiation of ESCs and iPSCs

The first human ESC line was established from surplus human embryos by Dr. Thomson's group in 1998 [7], which is 17 years after the successful isolation of mouse ESC line from the inner cell mass of early mouse embryos in 1981 [8], because of the different characteristics and growth requirement between human and mouse ESC cells. Derived from early embryos, ESCs exhibit two important properties: (1) they can divide and self-renew indefinitely when kept in proper conditions; (2) they are pluripotent and thus have the potential to differentiate into all cell types of the three germ layers, including cardiac myocytes [9, 10]. The iPSC technique was invented in 2006 by Takahashi and Yamanaka [11] who discovered that ESC-like, pluripotent stem cells can be derived from differentiated fibroblasts by viral expression of four transcription factors—Oct4, Sox2, Klf4, and cMyc—that are important for maintenance of ESC pluripotency. Like ESCs, human iPSCs can give rise to all cell types found in the human body, including cardiac myocytes [12, 13]. Unlike ESCs, iPSCs are generated without compromising human embryos, thus minimizing the ethical concerns. In addition, iPSCs can be derived from individual patients, making it possible to generate patient-specific iPSCs (and iPSC-derived cardiac myocytes) for personalized medicine research.

Most protocols for cardiac differentiation were established for ESCs and have been found to be largely applicable for iPSCs as well [13–15]. The traditional embryoid body (EB) method employs serum-driven spontaneous differentiation of ESCs and iPSCs into different germ layer cells, in which cardiac myocytes can be found [16]. This protocol does not require expensive recombinant protein factors but has a low efficiency for generating cardiac myocytes (<5%). The second generation of cardiac differentiation protocols employ recombinant protein factors for stage-specific activation/inhibition of signaling pathways, to recapitulate the temporal pathway activities in embryonic heart development. Specifically, Activin A and BMP4 (bone morphogenetic protein 4) are included in cell culture at the early stage to induce mesoderm, which is followed by addition of Dkk1 (a Wnt inhibitor) and VEGF (vascular endothelin growth factor) to promote cardiac lineage specification [17–19]. These directed cardiac differentiation protocols are highly efficient and capable of generating >70% cardiomyocytes (based on cTnT^+^ cells) but require expensive recombinant proteins, show significant batch-to-batch variation, and are labor-intensive especially when the EB formation is involved in the early stage [18]. The third generation of cardiac differentiation protocols only require the temporal modulation of the Wnt signaling pathway: activation at the early stage (e.g., with CHIR-99021) and inhibition (e.g., with Wnt-C59) at the late stage [20–22]. These protocols are based on monolayer cell culture, do not require expensive protein factors, and generate cardiomyocytes at a high efficiency with small molecules alone.

### 2.2. Direct Cellular Reprogramming

In 2010, Dr. Srivastava's group discovered that* de novo* cardiomyocytes can be generated by direct reprogramming of cultured mouse fibroblasts with 3 transcription factors (Gata4, Mef2c, and Tbx5) without the iPSC step [23]. Subsequent studies demonstrated direct cardiac reprogramming in animal hearts and such* in vivo* reprogramming has a higher efficiency than in cultured cells [24, 25]. Wang et al. investigated the effect of factor stoichiometry (Gata4, Mef2c, and Tbx5) on cardiac reprogramming and demonstrated that a higher level of Mef2c in combination with lower levels of Gata4 and Tbx5 increases reprogramming efficiency and promotes myocyte maturation [26]. A recent study by Zhou et al. identified Bmi1 as a key epigenetic barrier to cardiac reprogramming; inhibition of Bmi1 increases reprogramming efficiency and enhances the functional maturation of induced cardiomyocytes; Bmi1 inhibition can also substitute for Gata4 during the cardiac reprogramming [27]. Reprogramming of human fibroblasts into cardiomyocytes has been demonstrated, but additional factors (ESRRG and MESP1) are required [28]. In addition, different combinations of transcription factors and microRNAs have also been reported for direct reprogramming of somatic cells into cardiomyocytes with improved efficiency [29–32]. For example, Song et al. showed that a combination of four transcription factors (Hand2, Gata4, Mef2c, and Tbx5) reprograms mouse tail-tip and cardiac fibroblasts into functional cardiomyocytes in cell culture [30]. Jayawardena et al. demonstrated that transient transfection of microRNA-1, -133, -208, and -499 is also capable of reprogramming fibroblasts into cardiomyocyte* in vitro* [29].

Most recently, Dr. Ding's group has established a protocol that does not require transcription factors but uses only small molecules to reprogram human fibroblasts to cardiomyocytes [33]. This study is based on a cell activation and signaling-directed (CASD) reprogramming paradigm. The authors treated fibroblasts with chemicals to induce a progenitor fate in them and then applied cardiogenic molecules to differentiate the cells into mature cardiomyocytes. In sharp contrast to the transcription factor-mediated cardiac reprogramming that directly converts fibroblasts into cardiomyocytes, this method involves an early progenitor stage and the subsequent differentiation process. The advantage of direct cardiac reprogramming is that it does not involve the pluripotent stage and thus minimizes the risk of tumorigenesis after cardiomyocyte transplantation into host hearts. In addition, direct reprogramming can be performed* in situ* (inside the hearts) [6] and thus can avoid the issues of low survival, retention, and integration with host tissue, which are concerns for the cell transplantation strategy when iPSC- or ESC-derived cardiomyocytes are used. However, the efficiency of cardiomyocyte generation with direct reprogramming is much lower than that of cardiac differentiation of ESCs or iPSCs with current methods; the self-renewal property of ESCs and iPSCs also allows large scale cardiomyocyte generation.

## 3. Maturation of* De Novo* Human Cardiac Myocytes

Mature and immature cardiomyocytes have distinct properties in size, morphology, metabolism, and physiology. Although recent advances in technology have markedly improved the efficiency for* de novo* cardiomyocyte generation from pluripotent stem cells, the obtained cells exhibit an immature phenotype when maintained in standard culture [34, 35]. For example, hESC-derived cardiomyocytes typically have a small size (membrane capacity 18 pF versus 150 pF in adult cardiomyocytes) and a circular or irregular shape (versus rod-shape in adult), exhibit no T-tubules (present in adult and critical for excitation-contraction coupling), and use glucose as the primary metabolic substrate (versus fatty acid in adult), a more depolarized resting membrane potential (−60 mV versus −90 mV in adult), and a much slower action potential upstroke maximum velocity (50 V/s versus 250 V/s in adult) [35]. A study of the transcriptional landscape of cardiomyocyte maturation revealed marked shifts in cardiac gene expression during maturation and suggested that the immaturity of ESC-derived cardiomyocytes may result from dysregulation of critical transcription factors [36]. This immature phenotype represents a major challenge for the use of* de novo* cardiomyocytes in heart research, especially for disease modeling and drug testing for adult-onset heart disease. Therefore, much effort has been made to find ways to induce maturation of* de novo* cardiomyocytes (Table 1).

### 3.1. Prolonged Cell Culture

Maturation is a natural development process, and therefore increasing the culture duration has become an obvious strategy to promote maturation of* de novo* cardiomyocytes. Sartiani et al. reported that maintaining hESC-derived cardiomyocytes in culture for 3 months promoted electrical maturation of the cells, such as increased current densities for *I*_to1_ (transient outward potassium current) and *I*_K1_ (inward rectifier potassium current) [37]. In addition, Kadota et al. demonstrated a time-dependent increase in the conduction velocity in a monolayer of hESC-derived cardiomyocytes cultured for 30 days [38]. Lundy et al. showed that long-term culture (80–120 days) of hESC- and hiPSC-derived cardiomyocytes induced a more adult-like morphology and subcellular structure, including increased cell size, increased myofibril density, a better-organized sarcomere structure, and higher percentage of multinucleated cells [39]. Moreover, these long-term cultured hESC- and hiPSC-derived cardiomyocytes also exhibit more mature calcium handling and electrophysiological properties [39]. In summary, maintaining cardiomyocytes in a prolonged culture is a proven strategy for cell maturation, but it is both time- and labor-intensive. Therefore, alternative approaches to accelerate cardiomyocyte maturation have been explored.

### 3.2. Electrical Stimulation

hESC- and hiPSC-derived cardiomyocyte cultures can beat spontaneously. Nunes et al. showed that forcing hESC-derived cardiomyocytes in 3-dimensional culture to beat faster at 3–6 Hz (180–360 beats/min) with electrical pacing markedly promoted cardiomyocyte maturation, with organized myofibril structure and improved conduction velocity, as well as more adult-like calcium handling and electrophysiological properties [40]. Chan et al. reported that electrical stimulation of hESC-derived cardiomyocytes at 1 Hz increased expression of cardiac-specific genes (such as* SCN5A, MLC2V*, and* Kv4.3*), and a higher percentage of cardiomyocytes showed longer action potential duration, but electrical stimulation did not affect resting membrane potential or action potential upstroke velocity [41]. Hirt et al. reported that pacing heart tissues engineered with hESC-derived cardiomyocytes at 2 Hz led to enhanced contractile force [42].

### 3.3. Mechanical Stretch

Földes et al. reported that exposure of hESC-derived cardiomyocytes to cyclic mechanical stretch for 24 hours led to a 1.6-fold increase in cell size, increased mRNA levels of cardiac-specific genes (*αMHC, βMHC*, and* ANF*), and better-organized sarcomere structure [43]. Mihic et al. showed that cyclic stretch of 3D cultured hESC-cardiomyocytes increased percentage of cTnT^+^ cells, led to greater cell elongation, and enhanced connexin expression at cell junctions, as well as leading to more mature calcium handling [44].

### 3.4. Adrenergic Receptor Stimulation

Földes et al. reported that treatment of hESC-derived cardiomyocytes with phenylephrine (an *α*-adrenoceptor agonist) for 48 hours led to a 1.8-fold increase in cell size and better-organized sarcomere structure [43].

### 3.5. Thyroid Hormone Stimulation

Thyroid hormone plays a critical role in cardiac development [45]. Lee et al. reported that triiodothyronine (T3) treatment of mouse ESC-derived cardiomyocytes for 7 days increased expressions of cardiac-specific genes (such as* Nkx2.5* and* MLC2v*) and led to a more adult-like action potential profile and calcium handing [46]. Similarly, Yang et al. found that T3 treatment of human iPSC-derived cardiomyocytes for 7 days resulted in increased cell size, a 2-fold increase of single cell contractile force associated with faster calcium transient kinetics [47].

### 3.6. MicroRNA Overexpression

Fu et al. reported that overexpression of microRNA-1 (miR-1, which is highly expressed in adult cardiomyocytes) in hESC-derived cardiomyocytes led to a more mature-like (more hyperpolarized) resting membrane potential associated with increased potassium currents, as well as more mature calcium transient kinetics [48].

### 3.7. Summary

All the above studies have limitations: no single strategy can solve all the aspects of cell immaturity (Table 1). For example, electrical stimulation can improve functional maturation (action potential, calcium transient, and contraction force), while mechanical stretch increases cell size and sarcomere structure. Therefore, it seems that combination of such strategies may be a better solution for generation of mature cardiac myocytes.

## 4. Cardiac Subtype Myocyte: Heterogeneity of* De Novo* Cardiac Myocytes

The heart is composed of different cardiomyocytes (ventricular, atrial, and pacemaker myocytes) with distinct properties corresponding with their function in different parts of the cardiac chambers [49]. Pluripotent stem cells (ESCs and iPSCs) have the potential to give rise to all types of cardiomyocytes, making them a good model system to study cardiac diseases that affect different parts of the heart. However, this also poses a major challenge, because* de novo* cardiomyocytes obtained with existing protocols from ESC, iPSCs [21], and direct reprogramming [33] are a mixture containing all types of cardiomyocytes. Cell transplantation into the patient ventricles for regenerative therapy of heart failure would require a pure population of ventricular myocytes, and the contamination of pacemaker cells in the cell product will bring the concern for cardiac arrhythmias after transplantation [50]. On the other hand, studies of atrial disease, such as atrial fibrillation, would need a pure population of* de novo* atrial myocytes for disease modeling and drug testing. A pure population of pacemaker myocytes is required for biological pacemaker therapy of bradycardias. Therefore, development of new methods that allow enrichment of specific subtypes of cardiomyocytes from ESC, iPSCs, or direct reprogramming has been a hot research topic (Table 2).

### 4.1. Atrial Myocytes

Zhang et al. reported that 95% of the cardiomyocytes from differentiating hESCs after treatment with Noggin and retinoic acid (RA) had atrial-like action potentials [51]. Devalla et al. reported that treatment of differentiating hESCs with retinoic acid (1 *μ*M) from day 4 to day 7 (after the mesoderm formation stage but before the cardiac progenitor stage) increased atrial-specific gene expression (such as* KCNA5, KCNJ3*, and* NPPA*) but decreased expression of ventricular-specific genes (such as* MYL2, IRX4*, and* HEY2*) [52]. In addition, RA treatment increased the percentage of atrial myocytes (based on action potential profiles) from 20% in control untreated group to 85% [52].

### 4.2. Ventricular Myocytes

Zhang et al. found that treatment of differentiating hESCs with Noggin and a RA inhibitor increased the expression of ventricular-specific genes (*IRX4* and* MLC2v*), and 83% of the obtained cardiomyocytes had ventricular-like action potentials [51]. Weng et al. reported a protocol that generates 93–100% ventricular myocytes from different hESC and hiPSC lines based on electrophysiological properties of the cardiomyocyte population [53]. This protocol employs the sequential additions of a ROCK inhibitor, BMP4, Activin A, and IWR-1 (a Wnt inhibitor) in the cell culture, and the resulting ventricular-like cardiomyocytes exhibited typical ventricular ionic currents (*I*_Na_, *I*_Ca,L_, *I*_Kr_, and *I*_KATP_) [53].

### 4.3. Pacemaker Myocytes

Kapoor et al. demonstrated that* de novo* cardiac pacemaker myocytes can be obtained by direct reprogramming of rodent ventricular myocytes via viral delivery of* Tbx18*, a transcription factor required for embryonic pacemaker tissue development [54]. The translational potential of this work is suggested by preclinical studies carried out with pigs demonstrating that this strategy can be translated to large animals [55]. Jung et al. reported that overexpression of* Tbx3* in mouse ESCs promoted their differentiation into pacemaker cells, and in combination of cardiac-specific promoter antibiotic selection, >80% of the obtained cardiomyocytes exhibited a pacemaker-like phenotype [56]. Similarly, Ionta et al. found that overexpression of* Shox2*, a transcription factor critical for embryonic pacemaker tissue development, in differentiating mouse ESCs resulted in upregulation of pacemaker-specific genes [57]. The obtained pacemaker-like cells exhibited enhanced automaticity and are able to pace adult rat hearts* in vivo* [57].

## 5. Summary

Techniques that allow the generation of* de novo* human cardiac myocytes have revolutionized cardiac research. It is now possible to study heart disease mechanisms and perform drug testing on* de novo* human cardiac myocytes in culture dishes. Current efforts are focused on improving existing technologies for efficient generation of mature cardiomyocytes that only contain the desired subtype myocytes. With the improved methodologies,* de novo* human cardiac myocytes would be a powerful tool for medical research.

## Figures and Tables

**Table 1:** Strategies to promote cardiomyocyte maturation.

Strategies	Approaches	Findings	References	Pros/cons
Prolonged cell culture	3-month culture	Increased *I*_to1_ and *I*_K1_	[37]	Simple protocol but time-consuming
	30-day culture	Increased conduction velocity	[38]	
	80–120-day culture	More adult-like morphology and subcellular structure	[39]	

Electrical stimulation	3–6 Hz pacing in 3D cultured hESC-CMs	Adult-like structure, calcium handling and electrical properties	[40]	Requires special equipment and optimization of stimulation protocol
	1 Hz in monolayer culture	Prolonged action potential, but no effects on resting membrane potential	[41]	
	2 Hz in 3D tissue	Increased contractile force	[42]	

Mechanical stretch	Cyclic stretch	Increased cell size, better sarcomere structure	[43]	Requires special equipment and optimization of stimulation protocol
	Cyclic stretch	Cell elongation, increased connexin expression, more mature calcium handling	[44]	

Adrenergic receptor stimulation	Phenylephrine, 48 h	Increased cell size, better sarcomere structure	[43]	Simple protocol, easy to apply

Thyroid hormone stimulation	mESC-CMs: T3 for 7 days	Adult-like action potential profile and calcium handing	[46]	Simple protocol, easy to apply
	hiPSC-CMs: T3 for 7 days	Increased contractile force and faster calcium transient kinetics	[47]	

MicroRNA overexpression	miR-1	Hyperpolarized resting *E*_*m*_, faster Ca^2+^ transient kinetics	[48]	Requires introduction of foreign substance into cells

**Table 2:** Strategies for cardiac subtype myocytes generation.

Subtype cardiomyocytes	Approaches	Findings	References
Atrial myocytes	Noggin and retinoic acid	95% atrial-like cardiomyocytes	[51]
	Retinoic acid	Increased atrial-specific gene expression; 85% atrial myocytes in cell product	[52]

Ventricular myocytes	Noggin and retinoic acid pathway inhibitor	83% ventricular-like cardiomyocytes	[51]
	ROCK inhibitor, BMP4, Actvin A, and IWR-1	93–100% ventricular-like cardiomyocytes	[53]

Pacemaker myocytes	Tbx18-mediated direct programming of ventricular myocytes.	Pacemaker-like phenotype	[54, 55]
	Tbx3 overexpression in mouse ESCs	Increased yield of pacemaker-like cells	[56]
	Shox2 overexpression in mouse ESCs	More pacemaker-like cells, with enhanced biological pacemaker function	[57]
